# Reflections on the measurement of implementation constructs

**DOI:** 10.1177/26334895211020125

**Published:** 2021-06-11

**Authors:** Michel Wensing

**Affiliations:** Department of General Practice and Health Services Research, Heidelberg University Hospital, Heidelberg, Germany

**Keywords:** Measurement, validity, psychometric, implementation, dissemination, mental health

## Abstract

To advance research and practice, it is crucial to build on validated measures. A wide range of measures for implementation research were identified in seven systematic reviews conducted under the auspices of the project, “Advancing Implementation Science through Measure Development and Evaluation,” but many had unclear or limited measurement qualities. In this commentary, I suggest the psychometric paradigm of measurement validation may have to be reconsidered because many determinants and outcomes of interest are defined at higher levels of aggregation than the individual. Nonetheless, the practice of using non-validated measures should be reduced, and measurement validation research should be encouraged. Adaptation of existing measures to different domains, settings, and languages further adds to the need for validation research. Coordination of the development and validation of measures is required to avoid unneeded replication in some domains and lack of measures in others, and to take care that validation research remains instrumental to the purposes of implementation research and practice.

**Plain language abstract:** Many measures for implementation research have limited or unknown qualities. There is thus a need for better measures and targeted research is required to provide those. New studies should use measures of high-quality whenever possible.

Measures have a central role in research and practice. The analysis and interpretation of data can only be meaningful if the underlying measures are valid. New measures can offer fresh perspectives and lead to scientific breakthroughs. Under the auspices of an ambitious project, “Advancing Implementation Science through Measure Development and Evaluation” seven systematic reviews were conducted of published measures for implementation science, based on a shared study protocol (Lewis et al., 2018). The project was supported by the Society for Implementation Research and Collaboration (SIRC) and the National Institute of Mental Health (NIMH) in the United States. The reviews identified a range of measures, many of which had unclear or limited psychometric qualities. The published reviews comprise a Special Collection of *Implementation Research and Practice* entitled “Systematic Reviews of Methods to Measure Implementation Constructs.” This commentary will first briefly elaborate on the scope of the reviews, then discuss validation of measures in implementation science generally, and finally provide some recommendations for possible next steps.

## Scope of the reviews

The set of reviews is the most ambitious effort to review measures for implementation science to date. It adds to other reviews of measures in implementation science, such as a review focused on measures for implementation outcomes in physical health (Khadjesari et al., 2020) and a review of implementation-related measures in public health and community settings (Clinton-McHarg et al., 2016). These previous reviews also identified many measures and reported similar limitations regarding their psychometric qualities. The new set of reviews focuses on implementation research in behavioral and mental health care, based on literature searches up to May 2017. The identified measures may be useful in other health care settings, but this would require empirical testing. It seems likely though, that the conclusions broadly apply to implementation-related measures across all domains of health care.

The reviews were guided by the Consolidated Framework for Implementation Research (CFIR; Damschroder et al., 2009), which focuses on the implementation of practices by health professionals in health care organizations. The CFIR has been frequently applied to guide implementation research, but there are of course other theories, frameworks, and models in implementation science (Striffler et al., 2018). Measures for specific concepts may have been missed if they were not specified in the CFIR framework. For example, the range of constructs of context of implementation in the CFIR is more limited than in some other implementation science frameworks. The CFIR construct, “external policy and incentives” is a large box of items; in the Tailored Implementation for Chronic Disease (TICD; Flottorp et al., 2013) framework, these are specified as separate concepts such as political stability, corruption, malpractice liability, and economic constraint on the health care budget. It seems unlikely that measures of central concepts or broad domains were missed in the reviews because the CFIR overlaps substantially with other frameworks for implementation science, but measures of specific concepts not identified in the CFIR may have been missed.

It seems that all measures identified were questionnaires, mostly for health care professionals or health care managers. Qualitative measurement was explicitly excluded from the reviews, but other quantitative measures were apparently not found. Such measures exist, particularly for the measurement of professional performance, using health records or administrative databases. An example is data extraction from health records to document the adoption of evidence-based guidelines for depression and anxiety disorders (Smolders et al., 2009). In conclusion, the reviews are limited to questionnaire-based measures.

## Measurement validation in implementation science

The methodological assessment of the identified measures was based on the Psychometric and Pragmatic Evidence Rating Scale (PAPERS; Lewis et al., 2018). This checklist is firmly embedded in the psychometric paradigm (particularly in Classical Test Theory), which is often used for quantitative measures of individual knowledge, attitudes, abilities, cognitions, and personality traits. These unobservable phenomena within individuals are made tangible through the specification of items in questionnaires which are assumed to reflect underlying concepts. Although the psychometric approach to measurement is widely used in implementation science and other health research, reflections on the approach are warranted, and a few are offered here.

The psychometric approach takes *individuals* as the primary unit of analysis. Implementation indeed depends on the behavior of individuals (e.g., health care professionals), but it is the combined behaviors of individuals in a group or collective, which determines implementation outcomes such as adoption, fidelity, penetration, and sustainability. Furthermore, some determinants of behaviors are clearly related to individuals (e.g., behavioral intentions, or self-efficacy), but other determinants are characteristics of organizations (e.g., organizational readiness for change) or health care systems (e.g., financial reimbursement scheme). The psychometric approach to measurement may be less appropriate if the determinant or outcome of interest is defined at the level of higher levels of aggregation. For instance, team climate and team performance are not necessarily simple aggregations (e.g., mean values) of the perceptions of members in the team, as pointed out by Powell et al. (2021). In such situations, measurement qualities (e.g., reliability) of determinants and outcomes in implementation science may be more appropriately examined at higher aggregation levels than individuals.

A further reflection is related to the focus of implementation science on change of the *behaviors* of individuals (e.g., health care professionals). The effects of implementation strategies are often measured in terms of professional behaviors such as adherence to evidence-based guidance in clinical decisions, or in terms of health outcomes. These behaviors and health outcomes are observable, at least in principle, and there is consensus with respect to their nature (e.g., clinical guidance for health care professionals, or a biological model of disease). For instance, the previously mentioned study on depression and anxiety disorders (Smolders et al., 2009) operationalized guideline adherence for recommended treatment (i.e., a set of health professional behaviors) as an algorithm: documentation of psychological support or counseling or prescription of antidepressant medication or referral to a mental health specialist in patients with documented International Classification of Primary Care (ICPC) codes for depression (P03 and P76) or anxiety (P01 and P74). This algorithm essentially reflects consensus views, based on an evidence-based clinical guideline.

Such consensus views on the nature of phenomena can match with individual mental representations of clinicians and patients, but they can also be different. For instance, patient-reported symptoms may be consistent with a medical diagnosis, but not with patients’ mental representation of these symptoms. This implies that the psychometric approach to measurement validation may be less appropriate for concepts that relate to frameworks that are not individual mental representations. Some primary care physicians hold a mental model of depression than differs from the model that informs the clinical guideline, and therefore use other ICPC codes. Smolders et al. (2009) also included codes for episodes of feeling anxious/nervous/tense, acute stress, feeling/behaving irritable/angry, anxiety disorder, somatization disorder, or neurasthenia/surmenage. Strictly, this approach deviated from the clinical guideline, which did not support these coding practices or physicians’ underlying representations of depression. The study found that 80.8% of patients with a likely depression diagnosis (based on clinical interviews) and any documentation of depression diagnosis in the health records had received guideline-consistent care. This may reflect suboptimal guideline adherence, but to some extent it may also reflects that some physicians hold ideas about depression that differ from the prevailing clinical guideline.

Finally, the *feasibility* of measures is often crucial if measures are applied for practical purposes, such as in applied implementation research and practice. The psychometric approach does not take feasibility into account, yet feasibility sets constraints on the number and size of measures. In many applications, a balance between breadth and depth needs to be found. Breadth may be facilitated by the use of global measures, but this may come at the expense of the psychometric qualities. Global questions can be understood in different ways, which likely causes variations and reduces correlations with other measures. Measures of narrowly defined concepts are more likely to have good psychometric qualities but may result in unwieldy questionnaires. My experience in research suggests that a mix of global and specific measures is often optimal.

## Possible next steps

These reflections on the psychometric approach do not imply that standards for the validation of measures are not required. The range of methods may have to be widened, but it is crucial to consider the measurement qualities of measures in research and practice. The observed practice of one-time use of non-validated measures should be reduced, although it can probably not completely be avoided. In implementation science, measures often need to be tailored to target groups and settings, which implies that validated measures may be lacking. If validated measures are available, however, the reason for designing a new measure needs to be particularly convincing.

The systematic reviews show that the number of available measures varies widely across different concepts in the CFIR. It would be helpful to have mechanisms for the coordination of the development and validation of measures across studies and research teams in implementation research and practice. More coordinated programs of implementation science are needed to advance the field in many ways (Wensing & Grol, 2019), including the validation of measures. Coordination should help to reduce undesired duplication and address the absence of measures for relevant concepts. The results of the reviews suggest that current practice (research activities that are mainly driven by researchers’ interests and opportunities for funding) is insufficient to achieve this. For instance, several questionnaires for readiness for change in organizations have been developed (Weiner et al., 2020) and new measures are probably not needed.

There are many examples of existing measures which are adapted for use in other health care sectors, other countries, or other languages than originally. It needs consideration whether renewed validation research is required in such situations, and what approaches would be required. The translation into other languages usually implies a need for new validation research because a different language often comes with a different culture and society. Only few measures were developed in a multi-language research group from the very beginning. Even if the language remains the same, validation research may be required if the culture and context are substantially different from the original validation study. Validation research may also be needed, if a measure is applied in a different health care domain, or if it is administered in a different way (e.g., online instead of paper-based). In some other research domains, measurement validation has become a substantial enterprise (e.g., the validation of disease-specific quality-of-life measures). Validation research should remain instrumental to the ultimate purposes of implementation research and practice. Coordination may also be required to maintain a good balance between validation research and other types of implementation research.

## Conclusions

In implementation science, measurement validation is emerging, but still underdeveloped. To advance research and practice in the field, it is crucial to build on validated measures and avoid the use of non-validated measures as much as possible. Adaptation of existing measures to different domains and languages implies further need for validation research. Coordination of the development and validation of measures is required to avoid unneeded replication in some domains and lack of measures in others, and to take care that validation research remains instrumental to the purpose of implementation research and practice.
